# 2446. Effect of the Implementation of a Fluconazole Prophylaxis Protocol on the Incidence of Invasive Fungal Infection in High-Risk Neonates

**DOI:** 10.1093/ofid/ofad500.2064

**Published:** 2023-11-27

**Authors:** Patricia Marie D Isada, Marimel Pagcatipunan

**Affiliations:** Pasig City General Hospital, Manila, National Capital Region, Philippines; Philippine General Hospital, Manila, National Capital Region, Philippines

## Abstract

**Background:**

Invasive fungal infection (IFI) is one of the most important causes of morbidity and mortality in the neonatal intensive care unit. International societies have supported the use of fluconazole prophylaxis for neonates weighing < 1000 grams, but administration to other high-risk patients remains controversial. An institutional fluconazole prophylaxis guideline was implemented in December 2020 to include two other groups of neonates.

**Methods:**

One hundred thirty infants who were admitted at the NICU from January 2020 to January 2022 and fulfilled one of the following criteria: weight of < 1000 grams (Group A), weight of ≤1500 grams and on broad-spectrum antibiotics (Group B), or weight of >1500 grams and on prolonged nil per os (NPO) (Group C) were included in the study. They were divided into two groups: those who were born prior to the implementation of the protocol (January to December 2020) and not given fluconazole prophylaxis, and those born after (January 2021 to January 2022) and given prophylaxis. The primary outcome was the development of IFI.

**Results:**

A total of 130 infants were included in this study with 21 (5 control, 16 prophylaxis), 69 (28 control, 41 prophylaxis), and 40 (16 control, 24 prophylaxis) patients in Groups A, B, and C, respectively. There were no significant differences in the development of IFI among the groups (Group A: control - 40%, prophylaxis - 62%; Group B: control - 21.4%, prophylaxis - 22%; Group C: control - 29.2%, prophylaxis 31.5%) with a p-value of > 0.05. The presence of a central vascular access line, endotracheal tube, placement on NPO, histamine H2-blockers, and necrotizing enterocolitis were associated with the development of IFI. *Candida parapsilosis* comprised 60% of isolates. All isolates had 100% susceptibility to fluconazole. Jaundice developed in 3.7% of patients given fluconazole, with elevations in AST, ALT, and TB.

Incidence of fungal infection in high-risk neonates

**Figure:**
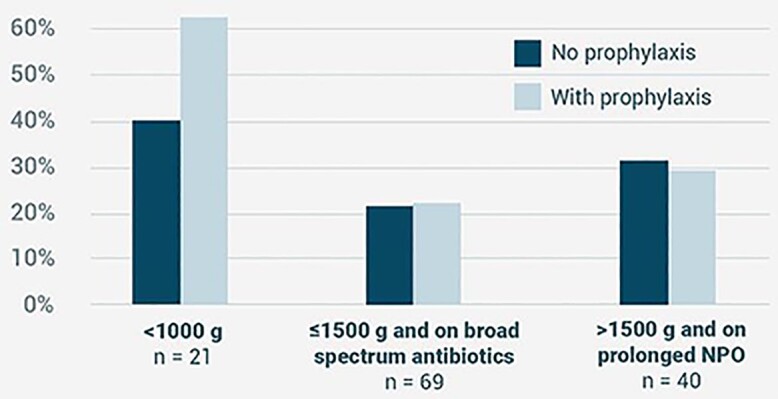

There were no significant differences in the development of IFI among the three high-risk groups, with p-value of > 0.05.

**Conclusion:**

Invasive fungal infection remains a heavy burden to high-risk neonates. The protocol at present does not significantly prevent the occurrence of IFI. There is a need to revise the protocol to include the presence of a combination of multiple risk factors (central line, endotracheal tube, prolonged NPO, H2-blockers, and necrotizing enterocolitis) before initiating fluconazole prophylaxis.

**Disclosures:**

**All Authors**: No reported disclosures

